# A Comprehensive Overview of Epidemiology, Pathogenesis and the Management of Herpes Labialis

**DOI:** 10.3390/v15010225

**Published:** 2023-01-13

**Authors:** Divya Gopinath, Kim Hoe Koe, Mari Kannan Maharajan, Swagatika Panda

**Affiliations:** 1 Basic Medical and Dental Sciences Department, Ajman University, Ajman P.O. Box 346, United Arab Emirates; 2 Centre of Medical and Bio-Allied Health Sciences Research, Ajman University, Ajman P.O. Box 346, United Arab Emirates; 3 School of Postgraduate Studies, International Medical University, Kuala Lumpur 57000, Malaysia; 4 School of Pharmacy, University of Nottingham Malaysia, Semenyih 43500, Malaysia; 5 Department of Oral Pathology and Microbiology, Institute of Dental Sciences, Siksha‘O’Anusandhan Deemed to be University, Bhubaneswar 751030, India

**Keywords:** herpes labialis, antiviral agents, treatments, prevention, management

## Abstract

Herpes labialis remains exceedingly prevalent and is one of the most common human viral infections throughout the world. Recurrent herpes labialis evolves from the initial viral infection by herpes simplex virus type 1 (HSV-1) which subsequently presents with or without symptoms. Reactivation of this virus is triggered by psychosocial factors such as stress, febrile environment, ultraviolet light susceptibility, or specific dietary inadequacy. This virus infection is also characterized by uninterrupted transitions between chronic-latent and acute-recurrent phases, allowing the virus to opportunistically avoid immunity and warrant the transmission to other vulnerable hosts simultaneously. This review comprehensively evaluates the current evidence on epidemiology, pathogenesis, transmission modes, clinical manifestations, and current management options of herpes labialis infections.

## 1. Introduction

Herpes labialis is an infection with herpes simplex virus type 1 (HSV-1) with initial episodes presenting as asymptomatic or symptomatic small blisters or sores on the skin near the site of infection. When the initial infection heals, the virus spreads to sensory nerve cells, where it remains dormant until reactivation occurs [1]. Recurrence of labial HSV is typically triggered by factors such as stress, trauma from surgery, menstruation or hormonal shifts in women, hyperthermia or infectious febrile ailments, sunlight or ultraviolet rays, and certain drugs, such as corticosteroids, with substantial psychosocial consequences to patients enduring persistent outbreaks [2].

The regularity of these recurrent outbreaks varies from one individual to another where the infection is generally contagious during times of active replication, but can also spread when symptoms are absent [1]. The disease progression in recurrent herpes simplex labialis (HSL) typically incorporates multiple stages. During the precursor stage, perceptions of pain, tingling, or burning may transpire in the affected area, followed by the development of vesicles. Rupturing of the vesicle leads to soft scab formation which is subsequently replaced by a hard scab. Periodically, the scab abates and falls off, allowing the lesion to completely heal without scarring. Throughout this healing process, symptoms such as pain and discomfort ensue, and complete healing may require seven to ten days [1,2,3,4].

## 2. Epidemiology

Oral HSV-1 infection continually remains exceedingly prevalent and is one of the most common human viral infections throughout the world. According to WHO, an estimated 3.7 billion people under age 50 (67%) have HSV-1 infection globally [5]. In the United States of America (USA), HSV-1 affects 57% to 80% of adults [6], while in Asia, the numbers are essentially high for adults (75%) especially those from low socioeconomic standings, and children (50%) due to epidemiological shifts, showing decreased seroprevalence in the young cohorts [7]. A reported estimate in 2016 revealed that around 3.583 billion were infected with oral HSV totaling a prevalence of 63.6% (95% uncertainty interval (UI): UI: 59.0–66.0) and the majority come from Southeast Asia region followed by the Western Pacific region [8]. It has been reported that recurrent herpes labialis (RHL) affects about one-third of the population in the USA and presents as inflamed/painful oral lesions with conditional distress. Some patients usually encounter up to six episodes per year which can be troublesome for patients and their families [6,9]. Overall, the average incidence of RHL is about 1.6 per 1000 patients each year with a prevalence of 2.5 per 1000 patients but varies significantly between countries and different communities, including more frequency in females where an estimated one-third of all affected patients experience at least one relapse per year [9]. In a similar study in Sweden, the lifetime encounters with symptomatic herpes labialis among 3597 participants were about 40% [10]. A cross-sectional survey among American college students reported HSV-1 antibodies’ prevalence of 37.2% in fresh graduates and 46.1% in final-year students, implicating a reduction in seroprevalence of HSV-1 from 62.0% (1988 to 1994) to 57.7% (1999 to 2004) [11] due to awareness.

## 3. Viral Pathogenesis

### 3.1. Pathogenetic or Symbiotic?

The natural history of oral herpes is described as human–virus symbiosis which converts reversibly from mutualism to parasitism in a balanced way and this co-existence occurs between superiorly co-evolved microorganisms with humans [12,13,14,15]. While symbiosis implies close acquaintance between two species that mutually provide benefits to both, the sophisticated nature of the virus to invade the host and evade the immune-responsive defense thus inflicting chronic infections suggests a “war-metaphor” which connotes more to a parasitic association where only the microorganism is provided with the benefit. Depending on the risk factors involved in the recurrence of HSV-1 oral infections, it is crucial to understand that there are no pure parasitic or mutualistic relationships between humans and HSV-1 [12].

### 3.2. Transmission & Infection

The majority of the primary infections result from direct exposure to bodily fluids such as saliva or exudates of progressive lesions, and proximate contact with lesions of infected individuals [16]. In addition, the transmission of the virus can also occur via kissing or sharing of towels/utensils. Usually, the primary infection emerges between 2 to 20 days following contact [5]. Approximately 2–10% of these infections also involve asymptomatic patients who periodically undergo HSV shedding in their saliva which is profound in immunocompromised individuals with an estimate of 38–40% [4,5,7,16]. Extensively, the asymptomatic HSV shedding is primarily reflected throughout the prodrome phase after infection, and 60% of these patients may not proceed to vesicle development even with the common symptoms of fever and pain [17].

At the contact site, HSV-1 primary infections are initiated after penetration of the tissue by the viruses and fusion of the viral envelope with the epidermal and dermal skin membranes (cellular and mucosal) [16]. Infection of the skin or the mucosa is accompanied by inflammation and tissue damage, causing the characteristic herpes blisters. Following efficient replication in epithelial cells, HSV reaches nerve endings of peripheral neurons and undergoes retrograde transport to the neuronal cell body [18]. Due to the breach of the immune system, the host cell’s retaliation to this viral suppression is disrupted, consequently evading immune responses with the complex reciprocation of the virion protein also known as “virion host shutoff” [16].

### 3.3. Latency and Reactivation

HSV often results in either lytic or latent infection. There is minimal gene expression and no viral particle generation during latency. The viral genome is capable of reactivation, which results in the creation of infectious virions in response to the right stimuli [19,20,21]. Sensory neurons harboring the virus contain nuclear transcripts known as latency-associated transcripts (LATs), responsible for modifying viral genome chromatins which generate a stable but reversible silencing of the HSV genome [21,22,23]. LATs are approximately 8.3 kb long noncoding RNA that expresses in the nucleus of latently infected cells [21,22,23,24,25]. Under latent conditions, LATs remain highly expressed and silence the immediate early (IE) gene expression, which are major genes in the lytic cycle [24,25,26]. LATs have a role in protecting the latently infected neurons from undergoing apoptosis in the epigenetic modification of histones to regulate the expression of the latent genes [27].

The HSV infection in the body of neurons usually results in infectious viral particles, while infection at the axons fails to produce infectious particles, especially when the infectious titer is low. However, it has been shown that axonal infection can lead to the production of an infectious virus if the cell body area is added with a helper virus with VP16, a tegument protein [28,29]. Moreover, it has been demonstrated that low levels of tegument proteins VP16 and ICP0 might lead to incompetent expression of genes leading to latency in neurons [29]. ICP0 also affects modifications of histone as well as expression of several viral genes and thus may play a significant role in latency [30,31]. Another interesting concept states that dividing the HSV stages of infection into latent and lytic cycles is not completely true, as intermediate stages with variable levels of gene expression have been recorded by few studies [29,32].

Epigenetic modifications have also been shown to regulate the latency and reactivation. The naïve HSV DNA lacks histones and once it enters the host cell, histone modifications occur on the HSV genome, thus restricting important gene expressions [33]. During replication, some viral proteins have been shown to decrease heterochromatin levels and increase euchromatin to help in gene expression, whereas, in latency, the HSV genome shows constitutive heterochromatin marks [34,35].

Although silenced, this reservoir can be stimulated from the trigeminal sensory ganglia and cause subsequent recurrent clinical outbreaks as reactivation occurs via common stimuli such as stress, ultraviolet (UV) radiation, temperature fluctuations, surgical trauma or dental procedures, immunotherapy, light/laser therapy, or even hormonal changes [20]. New infectious virus particles are created during reactivation in these neurons. These particles move anterogradely to the skin or mucosa, generating classic herpes sores. The virus may potentially enter the CNS, including the brain, and produce encephalitis or meningitis in immunosuppressive patients [20,21,22,23,24]. Recent studies have demonstrated that HSV-2 reactivation is very common in the vaginal mucosa of asymptomatic patients [36,37]. Many investigations have supported the idea that the HSV 1 regularly reactivates in the trigeminal ganglia after events such as immunosuppression, peripheral infection, and even stress. An increasing body of research suggests that asymptomatic infections occur often, followed by latency in neurons, and such recurring “mild” HSV-1 infections in the brain are associated with persistent neuroinflammation [38,39]. Microglia play an important role in the immune response to neuronal infection in the brain. Microglial cells endure an abortive infection after HSV-1 infection, proceeded by a burst of pro-inflammatory cytokine and chemokine release, which activates other glial cells within the CNS and recruits immune cells including NK cells, DC cells, and T lymphocytes to the brain [38,39,40]. HSV-1-infected microglial cells have also been demonstrated to produce reactive oxygen species which are directly responsible for damage to neurons [38,39,40]. Furthermore, it has been demonstrated that microglia continued in an active state in the brain of HSV1-infected mice until thirty days after infection, despite the absence of active replication [38,39,40]. However, whether the microglia have any role in the reactivation of herpes labialis is not yet known.

### 3.4. Immune Response to HSV-1

The intrinsic and innate immune responses are quite effective at controlling HSV reactivation after latency and primary infection [41,42,43,44]. Additionally, they provide a powerful immune system response against HSV. Reactivation nonetheless happens and is extremely common in some people which is most likely as a result of a combination of viral evasion mechanisms and insufficient immunological control. IFNs, one of the primary cytokine families that inhibit HSV, are expressed as a result of the majority of the signaling cascades that toll-like receptors (TLRs) initiate upon recognizing HSV [41,42,43,44,45]. Mammals have 13 TLRs that have been identified, and the main ones that sense HSV are TLR2, 3, and 9 [41,42,43,44,45]. When HSV binds or fuses, TLR2 detects viral glycoproteins, TLR3 senses dsRNA produced as a consequence of HSV replication, and TLR9 detects HSV DNA [41,42,43,44,45]. Other cytoplasmic and nuclear sensors also find intermediates of HSV DNA and RNA. To control the virus, it is essential to detect HSV nucleic acids. Severe HSE is more common in those with defects in DNA sensors, the RNA sensor TLR3, or downstream signaling pathways [41,42,43,44,45].

During the early stages of infection, adaptive immune responses are also crucial [45,46,47,48,49,50,51]. Since HSV-specific T cells are seen in both active and healed lesions in patients as well as in infected sensory human ganglia, the role of T cells is very important. Immunocompetent people have a low level of HSV-specific T cells in their blood after the acute infection has cleared up [46,47,48,49,50]. When exposed to viral antigens, blood CD8 T lymphocytes specific for HSV produce plenty of cytolytic molecules and release IFNs [46,47,48,49,50]. Around 22 HSV-1 proteins, including enzymes and structural proteins, are recognized by human CD4 and CD8 T cells, respectively [47]. HSV-specific CD4 T cells express cytokines that are similar to T helper type 1 (Th1) and Th0 and are capable of cytolysis [48,49,50]. The adaptive immune response also controls latent HSV and the depletion of CD 8 T results in higher reactivation [45].

## 4. Clinical Manifestations

Clinical manifestations appear as cold sores and oral lesions are distinctly characterized [52]. The initial stage of RHL generally commences with prodromal symptoms such as stinging pain, tingling or burning sensations, chilling tenderness of the skin, and itchiness at reactivation sites [53]. This occurs up to 6 h in 46–60% of patients caused by abrupt viral duplication at sensory neuron terminals of the epidermis or mucosal layers [17]. Hence, antiviral treatment is favorably given at this early stage before lesions develop to a greater extent, due to the narrow therapeutic window of most antiviral drugs [53,54,55,56]. Vesicle ruptures form scalloped patterned ulcers with surrounding erythema [55,56]. Around 25% of facial recurrences stay at the papular stage with common prodromal symptoms [17]. The disease usually resolves within 10 to 14 days and occurs naturally [17]. If untreated, further complications and morbidity ensue, such as the development of ocular blemishes, encephalitis, and subsequent severity may lead to mortality [53,53].

## 5. Triggers and Risk Factors

One of the foremost common triggers for RHL is fever, and this led RHL to be named herpes febrilis due to its characteristics. RHL occurs three times more frequently in patients with febrile conditions compared to those without so. Feverish patients with herpes labialis developed blisters with triple effect [16,57]. In addition, the frequency of the recurrence could also coincide with the expression of numerous candidate genes, namely human chromosome-21 (C21) or the Cold Sore Susceptibility Gene-1 (CSSG-1) [58]. Previous studies revealed a total of 6 candidate genes influencing herpes susceptibility and highlighted C21 or CSSG-1 to actively link to symptoms such as fever and the development of blisters via an unknown mechanism [56,59].

Sunlight or UV light in particular also presents a major and common trigger factor for RHL. Prone individuals such as swimmers, fishermen, farmers or skiers have prolonged exposure to sunlight and typically present with lesions and vesicles at the vermillion border of lips after 3 to 5 days [16]. Spruance et al. demonstrated that intentional exposure to controlled dosages of UV radiation resulted in the development of lower lip lesions in 60% of HSV-1 seropositive [60]. Moreover, administration of prophylactic acyclovir into HSV-1 seropositive skiers eventually prevented reactivation of latent HSV-1 compared to those on placebo [61]. Ultraviolet-B-susceptible individuals have also been more prone to experience recurrent infections of herpes labialis [62].

The lack of healthy zinc levels in HSV-1 individuals was also found to cause prolonged periods of lesion episodes, as this particular element has consequential effects in aiding recovery and healing of viral lesions [63]. As an essential mineral component in driving the natural processes of cells, tissues, and organs, zinc plays a major role in wound healing by coordinating the regenerative processes of the human body such as membrane reparation, inflammation reduction, immunity-boosting, oxidative stress decrement, cell epithelialization, and the formation of fibroids or scars [64]. The defense system in boosting immunity involves the T-cell lymphocyte response, providing a stimulated but controlled cellular immunity to viruses, fungal, or pathogenic infections, and certain autoimmune or immunocompromised diseases. The presence of zinc drastically improves the number of effector and helper T-cells, subsequently forming antibody precursors and contributing to the suppressive functions of responsible cells [65,66]. Kamakura et al. observed that the upregulation of the zinc finger transcription cell protein called insulinoma-associated-1 factor showed an increase in immunity-driven phagocytic activities to halt viral duplication and strengthen the local immunity system [67]. As such, zinc supplementation is proven to show benefits to individuals suffering from herpes and impaired T-cell functioning due to zinc deficiency [65]. Topical treatments of zinc oxide creams combined with glycine can produce considerable facial and circumoral appearances of herpes infections with or without inversed side effects [64]. Additional crucial element is vitamin D which exerts similar immunomodulatory properties to zinc and low serum levels have been shown to be coincidental with the recurrence of cold sores; additional research is needed to discover the actual mechanism [68].

Another major contributor to triggering virus reactivation is the elevated levels of psychosocial stress [69,70]. A meta-analysis covering multiple psychosocial and emotional assessments reported reliable positive correlations between chronic stress and recurrence of HSV-1 infections with clinical symptoms [71]. It is believed that immune system suppression, which enables viruses to evade immune monitoring, is the mechanism through which these stress factors affect the severity and recurrences of HSV disease [71,72,73,74]. However, the many HSV-infected neuronal types, including sensory and autonomic neurons, express the receptors for the two main stress hormones, adrenaline, and cortisol, in a selective manner [73,74]. It is possible that autonomic neurons play a unique role in the stress-induced modulation of productive HSV infection because the stress hormones epinephrine and corticosterone have different effects on HSV DNA replication and the release of infectious virus progeny [73,74]. However, the exact mechanism is not yet known. CD8+ T cells have been shown to play an integral role in the suppression of modulated immune functions caused by psychological stress and in preventing latent virus reactivation [72].

Immunocompromised hosts have a longer duration of episodes and are prone to contagious spread and comorbidities at higher rates, especially those undergoing bone marrow or organ transplants, chemotherapy, or dialysis. Although herpetic keratitis or encephalitis occurs rarely, these complications result from severe or untreated HSV-1 infections [56]. The white blood cell (WBC) differential variable is particularly used to predict the severity of immunosuppression although a direct association with herpes virus reactivation is yet to be confirmed. In one study of Caucasian ethnicity, the decreased lymphocyte count was found to have an association with herpes labialis, suggesting that modifications in WBC differential values account for risks of HSV-1 reactivation [75]. In addition, HSV-1 infection is considered “atypical” in immunocompromised individuals as they exhibit a larger extent of damage and aggression compared to immunocompetent patients, slower recovery (median time to healing up to 28 days), and a higher degree of pain. Moreover, co-infections with CMV and other viruses are common in immunocompromised patients [76]. Very little is known about the existence of viruses responsible for infections in the oral cavity and their interactions with each other.

In terms of the significance of HSV-1 trigger factors instrumental to herpes symptoms, the patient’s age, sex, the environment of residency, or inappropriate lifestyle affecting health such as smoking, consuming alcohol, or taking illicit drugs were less likely to contribute when compared to perceived elevated levels of stress. There were unclear occurrences in multivariate study models and cross-sectional studies, with no conclusive evidence of the apparent association between these putative risk factors and RHL [69]. The commonly reported trigger factors are listed as Table 1.

## 6. Management Strategies

### 6.1. Conventional Therapies

One of the earliest “home remedy” and long-standing treatments effectively used by RHL patients is cryotherapy, where self-treatment using ice cubes provides instant relief from prodromal symptoms, including burning/tingling sensations, itchiness, and pain [77]. Lip moisturizers and balms were introduced to counter humidity, and rough environments with fluctuating temperature changes which affect sufficient lip hydration [78] as these trigger factors are very likely experienced in most populations exposed to extreme weather and long exposure under the sun. Sunscreens, especially for lips, were also popularized not only for moisturizing but to block UV radiation from sunlight in seasonal weather [78,79,80,81,82].

Natural remedies, including the use of aloe vera leaf extract to produce a topical therapeutic effect with anti-inflammatory, anti-bacterial, and antiviral properties, have been tried as well. Rezazadeh et al. showed that aloe vera in gel form inhibited significant growth of HSV-1 at infection sites without any toxic side effects, indicating a convenient and safe choice to treat itchy and painful HSV-1 blisters and lesions [81]. Furthermore, the use of silica gel as an alternative gained approval as it was effective in the treatment of RHL with fewer side effects and had faster onset of action [82].

Another early drug used was levamisole due to its parasitic and antiviral properties. Although few studies showed mild effectiveness, it was proven to be lacking substantial evidence in a double-blind controlled trial by Russell et al., which did not find any significant differences in reducing frequency or severity between groups taking levamisole and the control group [83]. When it comes to targeting the pain barrier and feverish symptoms, anti-inflammatory drugs, analgesics, and antipyretics, such as paracetamol, ibuprofen, as well as mefenamic acid, were considered as there was an apparent connection between anti-inflammatory mechanisms and viral inhibitory effects of the drugs. Experimental studies found inhibition of viral duplications, in addition to antiphlogistic and anti-rheumatic properties of these drugs, but to a lesser degree [84]. One potent non-steroidal anti-inflammatory drug (NSAID) studied was piroxicam which exerts antipyretic effects as well as exerts a directly active antiviral response on the HSV-1 infection due to its lipophilicity, thus making this agent suitable as a topical form of treatment for herpes virus infections [85]. Even local anesthetics, such as tetracaine and lignocaine, have been prescribed to relieve the pain and itchiness arising from cold sores and fever blisters [86].

Internal supplementations were also believed to have some prophylactic effects in RHL individuals. Some of the earliest studies included L-lysine monohydrochloride in 1000 mg daily doses. A double-blinded, placebo-controlled crossover study by Milman et al. that evaluated 65 patients with RHL showed no apparent effect on the healing rate in the lysine group of patients but showed significant improvements in being recurrence-free [87]. However, several other studies provided data suggesting lysine monohydrochloride salts were largely ineffective in treating acute cutaneous HSV-1 infections [88]. On the other hand, Thein and Hurt’s study evaluating the long-term success and prophylactic efficacy of lysine showed a significant reduction in recurrence rates at high serum lysine levels (more than 165 nmol/mL) [89]. Additionally, high doses of lysine monohydrochloride, 1000 to 3000 mg/day, were found to reduce the recurrence rate in a study by Szapary and Cirigliano, which also specified the cost-effectiveness and safety when compared to oral antiviral drugs [90]. A combination of lysine–arginine supplementations also showed good control in managing RHL even when the lysine content is increased without affecting the balance of the original compound, suggesting the safety of supplementation even at higher doses [91].

Zinc ions have been reported to disrupt viral multiplication, with oral concentrations of zinc gluconate providing approximately ten-fold zinc serum levels in immunocompromised individuals [65,92]. Contrastingly, a recent meta-analysis by Cunningham et al. suggested inconclusive and limited evidence of zinc oxide creams in the treatment of RHL but found it was still reliable in lowering pain threshold and symptom periods [93]. This is specifically the case in immunocompromised hosts and critically ill patients as they are unable to consume oral concentrations of zinc [65].

Although NSAIDs, analgesics, zinc-based creams, and aloe vera gels were recognized as over the counter (OTC) medications, topical Docosanol (Abreva) is another OTC approved by the Food and Drug Administration in treating cold sores topically. It is a long-chain 22-carbon, saturated primary alcohol which inhibits broad spectrum lipid-enfolded viruses such as HSV-1 [88]. Experimental studies initially stated that docosanol interferes with nucleus localization and viral multiplications, but further experiments highlighted that it disrupts viral coalition with host cells and treatment should begin as early as possible for maximal effect [94]. The safety profile is also well-established without risk of resistance or side effects, making it one of the choices in managing most herpesvirus infections with adequate efficacy [95]. The conventional therapies available are listed as Table 2.

### 6.2. Antivirals

#### 6.2.1. Acyclovir, Valacyclovir, Penciclovir, and Famciclovir

Therapeutics involving antivirals for recurrent HSV-1 infections include systemic and topical agents called virustatics (Table 3). The first virustatic, idoxuridine, existed in the mid-1950s as a topical application for non-specific herpesvirus, while vidarabine was licensed in 1978 as the systemic compound for treating encephalitis [96,97,98]. Towards the late 1970s and early 1980s, nucleoside virustatics were introduced and have been widely recognized as the most prescribed antiviral drugs for RHL, due to their safety and efficacy benefits [99]. The first of these landmark nucleoside inhibitors is the potent and selective Acyclovir (Zovirax) which disrupts DNA polymerase of herpes virus, followed by its generic alternative, prodrugs Valacyclovir and Famciclovir, and a resembling analog Penciclovir [96]. While there were more efficacy data available for oral antivirals in treating acute infections [90], expansive contributions continue to appear for topical antivirals such as 5% acyclovir as the first-line therapy for RHL and 1% penciclovir [82]. Although over the years numerous clinical trials conducted globally resulted in a new generation of antivirals for herpes infections, acyclovir remains the “gold standard” [96].

Another cream formulation commonly used is foscarnet (trisodium phosphonoformate) which selectively disrupts HSV-1 DNA polymerase activity, showing clinical efficacy in decreasing the size and development time of recurrent lesions [100]. Although foscarnet is employed exclusively to treat infections with cytomegalovirus (CMV), acyclovir-resistant herpes simplex virus (HSV) also tends to respond to this drug.

Structural analysis of acyclovir revealed that the molecule is almost identical to the acyclic DNA-base guanosine [82]. Like all nucleoside analogs, they are favorably cleaved by infected viral cells, where they undertake initial phosphorylation at 3000 times faster than normal unaffected cells via Tyrosine Kinases (TKs), the viral encoded enzymes [101,102]. With this attribute, acyclovir is effortlessly phosphorylated by these viral TKs compared to readily available cellular TKs, with maximum concentrations of the monophosphorylated drug in the infected cells compared to the normal uninfected cells [101,102]. Then it is converted to the active triphosphate configuration by cellular kinases to be incorporated into the elongated viral chain as opposed to normal cellular polymerase, leading to viral multiplication remission and chain termination [101]. This shows that acyclovir acts not only as a chain terminator but also as a competitive inhibitor for viral TK, while the acyclovir–triphosphate complex molecule becomes an imperious suicide inhibitor for the viral DNA polymerase, ceasing the activity of the exonuclease. As this cycle is repeated, HSV-1 DNA polymerase continues to add more nucleotides to the growing DNA chain until the excised nucleotides reach acyclovir again. The viral DNA polymerase is thus inactivated, and viral genomes are unable to reach full replication or form mature strains of HSV-1 [10].

#### 6.2.2. Problems of Bioavailability and Resistance Leading to Novel Approaches for Newer Antivirals

To overcome the inferior hydrophilic nature and poor oral bioavailability of acyclovir, the prodrug valacyclovir was introduced via valine esterification of acyclovir. With higher bioavailability, there was increased absorption, approximately 54% in the intestinal region, thus preventing wastage when oral doses are given [102,103]. Valacyclovir is converted back into active acyclovir and valine via metabolism in the liver and kidney by the protein-like enzyme biphenyl hydrolase where the esterified amino acid is cleaved from the molecule [103,104,105]. Another prodrug developed with the same objective of more bioavailability and higher water solubility was penciclovir with a swifter phosphorylation process and a longer half-life in triphosphate form than acyclovir [103]. However, penciclovir contains the 30-hydroxyl group on the acyclic side chain which limits the extension of the nucleoside chain, thus removing the chain termination ability as seen in acyclovir although still significantly inhibiting DNA polymerase [101]. As a result, oral penciclovir has a significantly lower bioavailability than acyclovir (1.5% vs. 5%) and a shorter half-life (2–2.5 h vs. 4–5 h) and due to this, penciclovir is preferably formulated as topical creams [104]. To rectify the poor oral bioavailability and short half-life of penciclovir, the newer prodrug famciclovir, the diacetyl 6-deoxy ester of penciclovir with improved oral uptake of around 73%, was introduced [101,104]. The new orally administered prodrug can be converted completely to penciclovir via oxidation at the 6-position by the hepatic enzyme cytosolic aldehyde oxidase following first-pass metabolism and initial deacetylation by intestinal esterases, leading to the same antiviral and pharmacokinetic properties as penciclovir [106]. A single daily dose of famciclovir is rapidly absorbed in the gastrointestinal tract, yielding approximately 77% bioavailability even without the presence of food [107,108].

The resistance toward antiviral nucleoside analogs arises from two mechanisms: mutated TK genes in viral cells and mutations in DNA polymerase, which inhibits DNA synthesis and interferes with antiviral activity. This tends to occur more in immunosuppressed individuals as compared to normal immunocompetent patients with frequent RHL attacks as reported by Mubareka et al. in their comprehensive review of 11 clinical trials [106]. Further studies on acyclovir showed that viral resistance resulted mainly from mutant TKs [103]. As viral TKs are essential for the conversion of acyclovir to the monophosphate form, their absence subsequently blocks host enzyme phosphorylation resulting from single and multiple mutations of these TKs [104]. This viral TK deficiency demonstrates an identical cross-resistance pattern among all the nucleoside analogs from acyclovir, valacyclovir, penciclovir, and famciclovir among immunocompetent individuals, but the higher prevalence among immunocompromised patients with severity depending on underlying diseases such as hematologic transplants, stem cell replacements or solid organ surgeries [107,108]. Thus, immunocompromised patients with a higher magnitude of suppression to their immune system encounter more frequent recurrences, longer periods of clinical symptoms, lesion sizes, and in more severe cases, become life-threatening [109].

Even to this day, there have been minimal clinical trials that directly compare oral or topical therapeutics for RHL outbreaks. Antivirals moderately reduce the time to healing and pain subsiding, with valacyclovir showing efficacy in these two preferred outcomes, famciclovir only at a specific dosage, while acyclovir was effective in decreasing only pain duration [110]. Essentially, treatment with antivirals should start as early as possible at the prodromal phase of herpes labialis [109].

A novel approach included the introduction of newer drugs, amenamevir (ASP2151) and pritelivir (BAY 57–1293), belonging to the helicase–primase inhibitor class, with the heterotrimer complex comprising accessory proteins UL5 helicase, UL52 primase, and UL8 subunit. As the absence of a eukaryotic homolog is mitigated by this helicase–primase complex, viral replication is blocked [109]. Early phase II clinical trials only involved animal models and HSV-2 infections, but further trials are required for understanding the clinical potential of these two promising drugs on HSV1 infections [111].

Another novel approach focused on new delivery systems which can provide more efficient drug administration to enhance skin penetration with unique vesicular carriers called ethosomes. They mainly contain phospholipids along with high content of ethanol and water. Ethanol mainly allows the fluid formation of lipids in ethosomal molecules and allows compact lipid arrangement in the skin barrier. Hence, to enhance skin penetration, ethosomes use these double effects of ethanol on lipid bilayers of the stratum corneum and the vesicle, permitting soft vesicles to penetrate the modified stratum corneum structure and gradually release the active drug in extended skin layers [112]. Studies by Horwitz et al. and Nainwal that focused on ethosomal delivery of acyclovir, showed significant improvement to all relevant clinical measurements compared to the pioneer Zovirax cream, thus formulated as the new Supra-Vir acyclovir cream using this ethosome technology [113,114].

Similarly, nanomedicine involving nanoparticles was proven effective for medicinal applications using particulate components with approximately 100 nm in size or lower. It was found to have lower toxicity and adverse effects while maintaining effectiveness in targeted treatments, where efficacy was enhanced with nanoparticle surface adjustments and preservation of structural properties among other biological, antiviral, antimicrobial, and pathogenic characteristics [115]. Hence, this approach led to an experimental polyethylene glycol-coated zinc oxide nanoparticle with acyclovir to potentiate systemic and local therapies to fight against HSV-1 infections. Initial findings showed some antiviral potential for HSV-1 infections in a cell culture model with low cytotoxicity but more clinical trials are required [116].

### 6.3. Emergence of Bioactive Natural Products

Although non-pharmaceutical approaches beginning with nutrient supplementation such as zinc, vitamin C, vitamin D, lysine, and low arginine diets have some potential benefits in the management of HSV-1 infections, results were mostly inconsistent and unreliable. Although the emergence of other natural remedies showed encouraging results in some clinical trials, most did not provide complete safety profiles or were assumed to be safe due to their natural identity. A comprehensive review by Münstedt provided essential evidence of numerous natural medicaments, such as L-lysine, lemon balm (Melissa officinalis), aloe vera extract, astragalus, bee propolis, elderberry, echinacea, cat’s claw, sage-rhubarb, sandalwood, tea tree oil, and witch hazel, in preventing illnesses and alleviating diseases [117]. Bee propolis as lip balms was proposed as an alternative to topical antiviral applications as one study confirmed its superiority and safety when used in cases where standard antivirals were not profoundly tolerated [118]. An open-label RCT using medical-grade kanuka honey with superior antiviral effects towards HSV labial occurrence highlighted that botanical compounds can be an alternative to acyclovir with a better safety profile [103,119].

Some of the established zinc-based creams synergistically enhanced in combination with herbal-based ingredients, including echinacea and goldenseal, resolved RHL outbreaks within three days in 40% of affected individuals [120]. With stronger antiviral activities and a simple mechanism of action potentiating prophylactic benefits on viral growth stages, these natural products were favorable because of the lower chance of developing resistance, lower toxicity, and price compared to antivirals [120]. Additional natural compounds, which have been tested at basic trial levels, include neem-based creams, beeswax, jojoba oil, shea butter, sesame oil, peppermint oil, and also coconut oil [119].

### 6.4. The Modern, Non-medicinal Future: Laser Therapy Immunotherapies and Probiotics

As the world moves forward in technological advancements and global opinion favoring a non-medicinal future, laser treatments, electrical stimulation, immunotherapies, and vaccinations carry a huge expectation in solving RHL. Based on the literature, there were no confirmed laser therapy modalities that can fully decimate virus replication and prevent recurrence with 100% success, but clinical trials did show a reduction in the pain and frequency of recurrences in almost all patients suffering from primary and secondary infections of herpes labialis [121]. A recent review concluded that although low-level laser therapy is superior to most antivirals in RHL therapy, the inconsistent study designs and unparallel clinical parameters hinder robust conclusions and hence, more standardization is warranted [122]. Another novel method is the transdermal electrical stimulator, especially in patients intolerant to antiviral agents [88]. Additionally, the topical immuno-sensitizer squaric acid dibutyl ester (SADBE) may help in the prevention of RHL outbreaks and reduce frequency rates by delaying hypersensitivity reactions [123]. A promising risk–benefit analysis of topical 2% SADBE by Chang et al. suggested that this approach may decrease the recurrence rate and severity of outbreaks, although it may take up to 3 months for the treatment effect [124]. Finally dendritic cell vaccines could be a ray of hope when other treatments seem to give no efficacious impact [125]. With the incorporation of HSV-1 recombinant glycoprotein D vaccines in activating HSV-1-specific immune responses, mononuclear cells will receive a decreased mitogenic reaction which allows normalization of the immune system, providing a balance in viral replication breakdown [126].

Altering the commensal microbiome indigenous to a specific body site has been tried and tested for the management of several diseases. It has been reported that the commensal microbiome primes the immune system and the microbiota can promote antiviral immunity [127,128]. A recent study suggested that gut microbiome can play a role in preventing HSV encephalitis via the gut–brain axis [129] and hence, probiotics are being explored for the management of encephalitis. The bacteriocin subtilosin generated by Bacillus species affects the late infectious phases of both HSV-1 and HSV-2 invitro [130]. The subtilosin, another enterocin from Enterococcus faecium, also suppresses the early infectious cycle of HSV-1 in vitro [127,130]. Whether the manipulation of the gut and oral microbiome prevents herpes labialis infection is yet to be explored.

## 7. Impacts on Quality of Life

### 7.1. Physical and Psychological Implications

Unintentionally, patients and treatment providers often resort to quick treatment for RHL without a complete assessment of viral infection severity, dissemination of risk factors, or even internal immunocompetency checks [2]. Hence, problems with antiviral resistance, absorption, distribution, metabolism, and elimination add to dispensable aspects in the quality of life of the patients which can be impactful depending on the severity and frequency of the infection. While most symptoms are often mild enough, the prolonged duration of the illness may become uncomfortable and traumatizing up to the brink of emotional impairment. This psychological impact may especially affect the young and self-conscious where facial appearances are a priority [56].

In combining both psychosocial methods for the prevention of recurrence and reducing psychological stress, outcomes may be favorable to both the patients and also healthcare professionals [110]. It is interesting to note considerable data connecting emotional distress or negative emotions to HSV epidemiology, where RHL can frequently occur due to triggering factors, including disgust and misery [131]. Constant sleep deprivation may cause lasting effects on the body, especially on skin functionality and integrity. With prolonged sleep deficit, stress can be elevated without incitement and affect the modulation of the hypothalamic–pituitary–adrenal axis in the body, thus increasing excessive glucocorticoid production to put the body in an immunocompromised state and triggering psychological effects to reactivate HSV-1 infections [132].

### 7.2. Lifestyle Modifications (Habits, Hygienic Considerations, Dietary)

Practical methods in lifestyle modifications can be convenient alternatives compared to substantially expensive treatments or risky therapies and these include prevention of prolonged exposure to sunlight, use of sunscreen, exercise, and relaxation methods to reduce emotional stress, healthy hygiene habits, and healthy and sufficient sleep [133]. All these adjustable lifestyle factors were further explored in a review of holistic dermatology techniques by Hu et al. who highlighted the interrelationship between healthy habits and dermatological health in mitigating the progressive nature of most viral skin infections [132]. Dietary adjustments can also include L-lysine [134], zinc, and vitamin C supplementation as they have been linked to the healing process. A combination of alcohol drinking and nutritional decline possess a serious influence on a weakened immune defense and thereby RHL recurrence, and hence, could be tired to be controlled by lifestyle modifications [132].

## 8. Conclusions

Seroprevalence data show that oral HSV-1 infection regularly transmits among vulnerable humans as the virus transition constantly happens from latent to recurrent stages. A variety of management regimens ranging from natural sources to potent oral/topical medications establish sufficient relief, although still few available agents offer only temporary relief and are unable to overcome bioavailability/resistance problems. In addition, the combination of psychosocial techniques to relieve stress, and adjusting lifestyle modifications reasonably may also have positive implications in quality-of-life aspects. More substantial research and consolidated evidence are needed to further associate viral infection/reactivation with exact triggers and development of prediction models for reactivation/recurrence.

## Figures and Tables

**Table 1:** Commonly reported trigger/risk factors for recurrent herpes labialis.

• Fever	• Orofacial fracture
• Flu	• Emotional/psychosocial stress
• Exposure to sun/UV light	• Upper respiratory issues
• Seasonal weather	• Immunosuppressive treatments
• Chapped lips/trauma	• Chemotherapy
• Fatigue	• Organ transplantations
• Lack of sleep	• Additional viral infections

**Table 2:** List of early preventive/management interventions [96].

• Ice cubes	• Anesthetics
• Lip balms/moisturizers	• Lysine
• Lip sunscreens	• Zinc and iron supplementations
• Aloe vera gel	• Vitamin D supplementations
• Silica gel	• Zinc-based creams
• Levamisole	• Docosanol
• Antipyretics	• Foscarnet
• Painkillers	

**Table 3:** Categorization of nucleoside analogs of antiviral drugs for various herpesvirus indications and targeted mechanisms [96,97].

Product (Brands Available)	Category	Targeted Mechanisms	Recommended Dosage/Routes	Indication/ Viral Spectrum
Acyclovir (Zovirax)	Purine analog	TK, DNA polymerase, chain termination with competitive dGTP	Oral: 5 × 200–800 mg (5–14 days) Topical: 5% ointment/cream	HSV, VZV, HCMV
Valacyclovir (Valtrex)	Purine analog, prodrug for ACV	TK, DNA polymerase	Oral: 2 × 500 mg^−1^ g (5–10 days)	HSV, VZV, HCMV
Famciclovir (Famvir)	Purine analog, prodrug for PCV	TK, DNA polymerase	Oral: 2 × 125–250 mg (5–10 days)	HSV, VZV
Penciclovir (Denavir/Vectavir)	Purine analog	TK, DNA polymerase, no chain termination but with competitive dGTP	Topical: 1% cream	HSV, VZV

Abbreviations: ACV = Acyclovir; VCV = Valacyclovir; FCV = Famciclovir; PCV = Penciclovir; TK = thymidine kinase; DNA = deoxyribonucleic acid; dGTP = deoxyguanosine triphosphate; HSV = herpes simplex virus; VZV = Varicella-zoster virus; HCMV = human cytomegalovirus.

## Data Availability

Not applicable.
